# Recurrent cutaneous necrotizing eosinophilic vasculitis: a case report and review of the literature

**DOI:** 10.1186/1746-1596-8-185

**Published:** 2013-11-07

**Authors:** Wenfei Li, Wang Cao, Haiyan Song, Yanxia Ciu, Xianmei Lu, Furen Zhang

**Affiliations:** 1 Shandong Provincial Hospital for Skin Diseases, Shandong University, Jinan 250022, China; 2 Shandong Provincial Institute of Dermatology and Venereology, Jinan 250022, China; 3 Department of Dermatology, Qianfoshan Hospital, Shandong University, Jinan 250014, China

**Keywords:** Vasculitis, eosinophilic, Recurrent, Ctaneous necrotizing, Corticosteroid

## Abstract

We report a case of recurrent cutaneous necrotizing eosinophilic vasculitis (RCNEV) in a 57-year-old male. The patient presented with papules and pruritus of the lower limbs of more than 1 month duration, and with angioedema and intensively pruritic, necrotizing lesions of the bilateral anterior tibias and feet for 2 weeks. Treatment with systemic corticosteroids was administered for 1 month, and resulted in a significant improvement. We also present a review of the pertinent literature and discuss the clinical features, histopathological features, and differentiation of RCNEV.

**Virtual Slides:**

The virtual slide(s) for this article can be found here: http://www.diagnosticpathology.diagnomx.eu/vs/2065600765102207

## Background

Recurrent cutaneous necrotizing eosinophilic vasculitis is a rare disease, which has clinical features of annular urticarial plaques, pruritic purpuric papules, angioedema, long course, chronic relapsing process, and an absence of any features of the systemic disease. Histopathological findings of RCNEV show necrotizing vasculitis of dermal small vessels with prominent eosinophilic infiltration. RCNEV was first reported by Chen, in 1994, and to the best of our knowledge there are only five patients with RCNEV described in the literature. Peripheral blood eosinophilia is a feature of many diseases such as hypereosinophilic syndrome, Wells syndrome, Churg-Strauss syndrome, and eosinophilic fasciitis. Compared with the aforementioned diseases, RCNEV has distinct pathological features of fibrinoid degeneration on small vessel walls and necrotizing vasculitis of dermal small vessels with prominent eosinophilic infiltration. Here, we report a case of RCNEV in a 57-year-old male, whose diagnosis was made using clinical, histopathological, and laboratory analysis results, and who was treated using systemic corticosteroids.

### Case presentation

**Figure 1:**
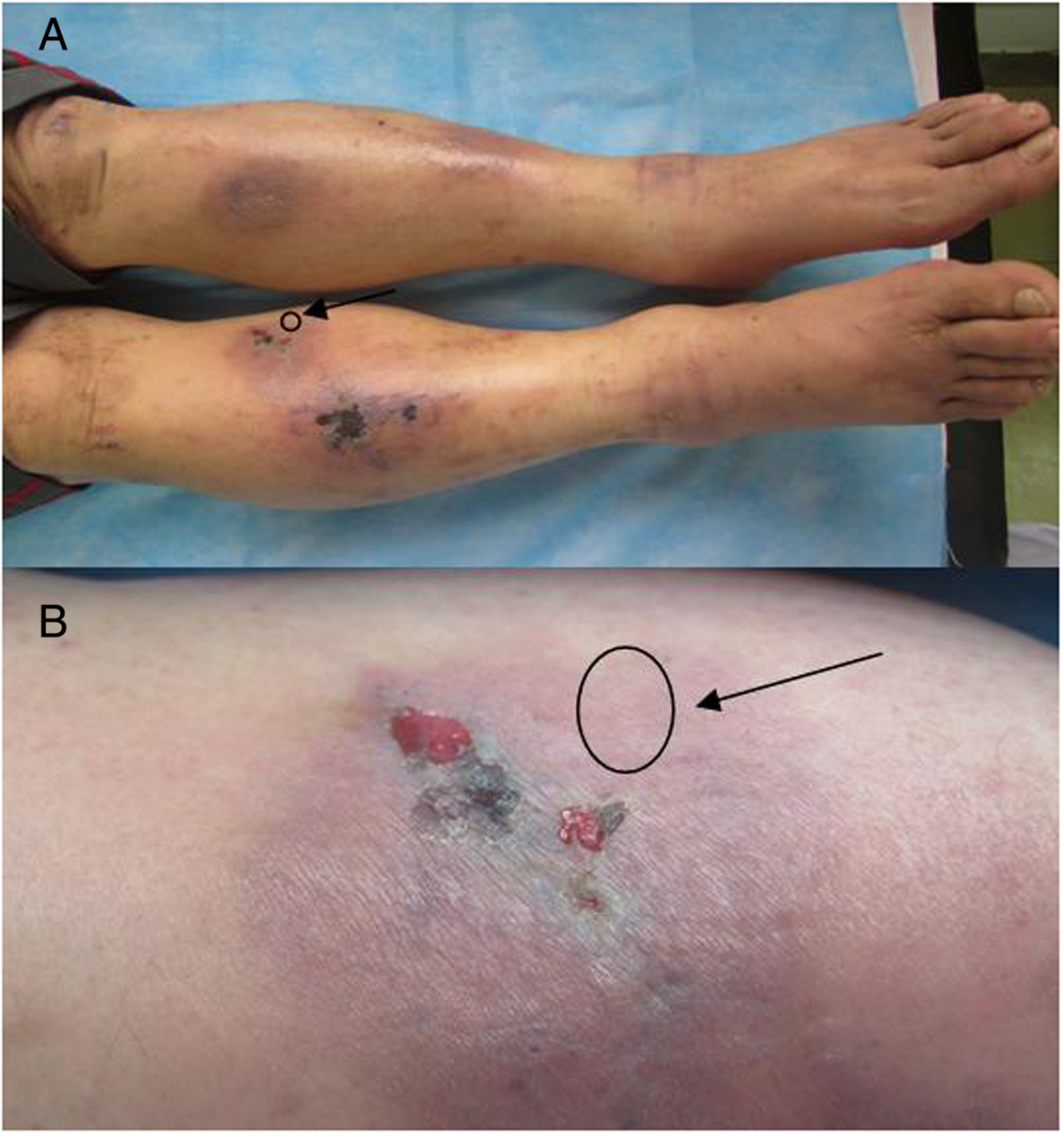
**Typical rash showing necrotizing lesions, plaques of purpuric angioedema, and excoriations predominantly localized on the lower limbs.** A skin biopsy was performed from the area indicated by the arrow. **A**: Necrotizing lesions and plaques of purpuric angioedema on the bilateral lower legs and the tops of feet. **B**: Excoriations on the plaque of purpuric angioedema.

A 57-year-old Chinese male complained of papules and pruritus of the lower limbs for more than 1 month, and angioedema with intensively pruritic, necrotizing lesions of the bilateral anterior tibias and feet for 2 weeks. Several needlepoint-sized papules appeared on his lower limbs, and the patient was treated for “eczema”. However, the skin lesions increased, with itchy needlepoint- to millet-sized papules appearing on the anterior tibias. Three weeks later, lesions of the lower extremities exacerbated, papules merged into purpuric plaques with angioedema, and some lesions became necrotic. Laboratory examinations and a skin biopsy were recommended. He was a farmer , and lived in a small village. He had no history of smoking, drinking and potential exposure to dust. No potential anomalies in nutrition. He did not have a family history of dermatoses, allergic rhinitis, or asthma. He denied a history of insect bites and drug eruption, but had more than five years history of psoriasis. Physical examination showed that necrotizing lesions, plaques of purpuric angioedema, and excoriation were predominantly localized on the lower limbs (Figure 1). He did not experience fever or weight loss, and his blood pressure was normal. Electrocardiogram, chest X-ray, abdominal ultrasound, and cranial computed tomography investigations indicated no cardiac, pulmonary, hepatic, splenic, nephritic, central nervous system, maxillary sinus, or other visceral organ involvement.

**Figure 2:**
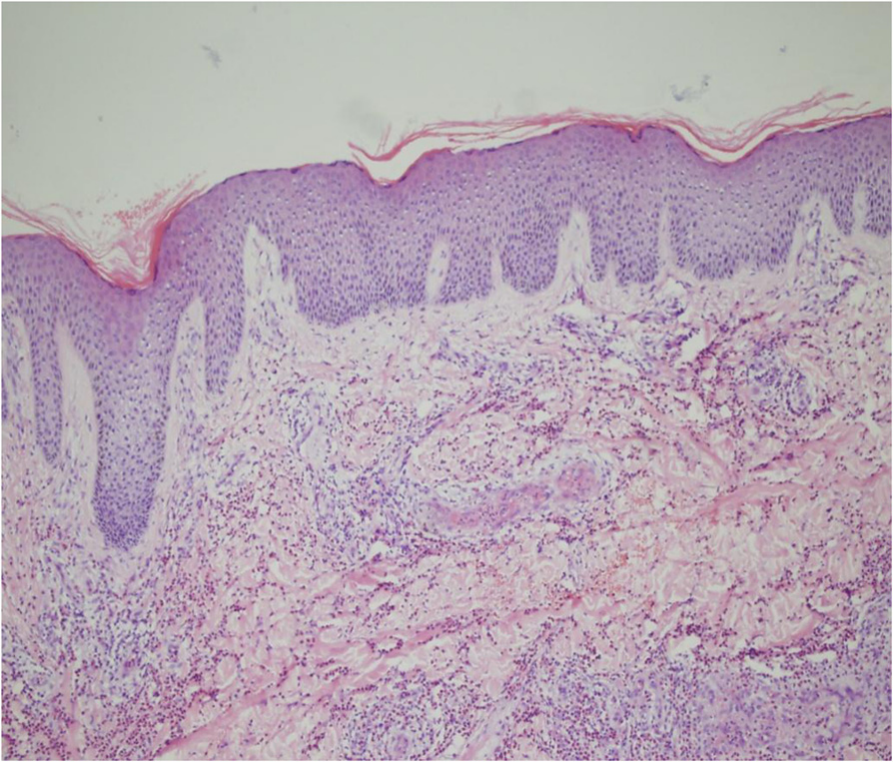
**Normal epidermis and the infiltration of numerous inflammatory cells in the upper and deep dermis.** Thickening of the vessel walls and infiltration of inflammatory cells perivascular and into vessel walls (H&E × 100).

**Figure 3:**
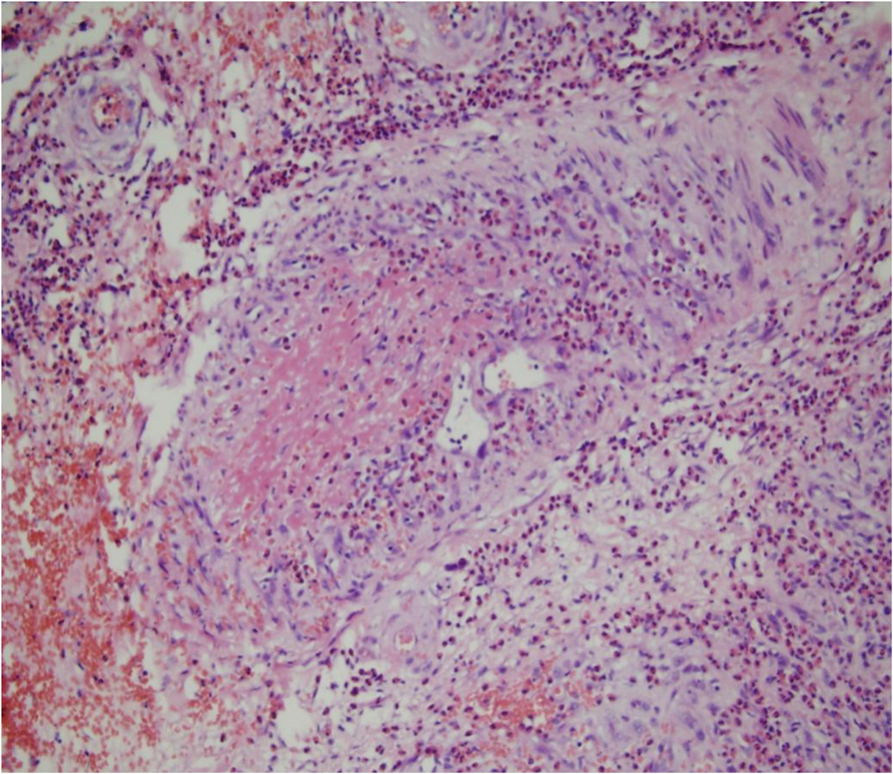
**The infiltration consists of numerous eosinophils and a few neutrophils perivascular, into vessel walls.** Numerous extravascular erythrocytes and fibrin thrombi in the lumens (H&E × 200).

**Figure 4:**
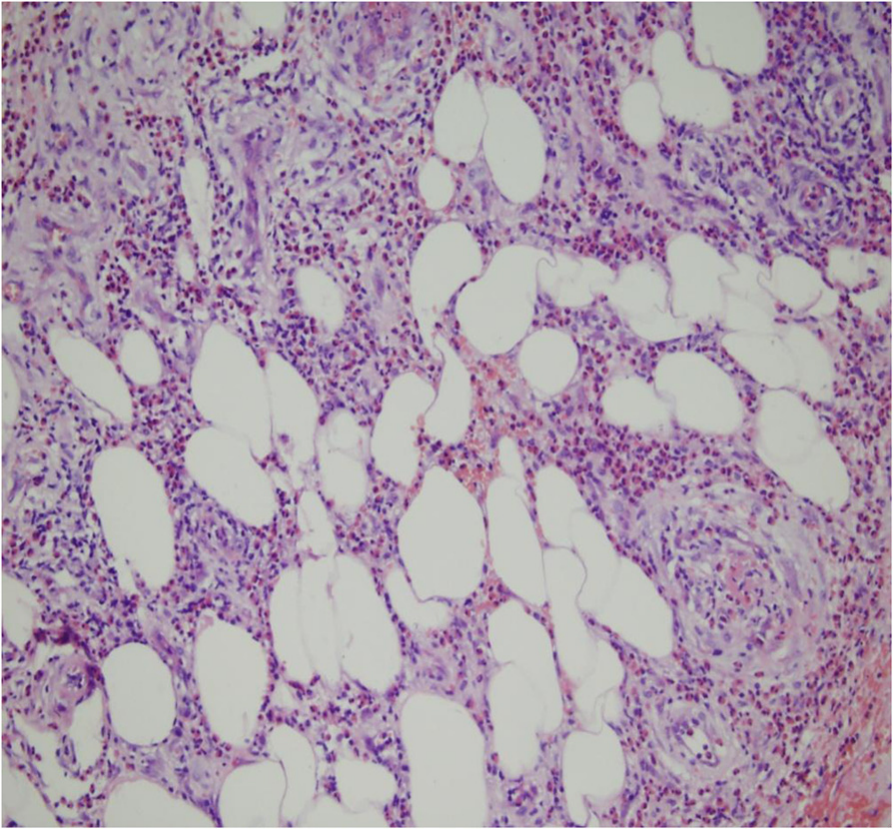
**The infiltration consists of numerous eosinophils and a few neutrophils perivascular, into vessel walls, and in the subcutaneous tissue.** Numerous extravascular erythrocytes and fibrin thrombi in the lumens (H&E × 200).

**Figure 5:**
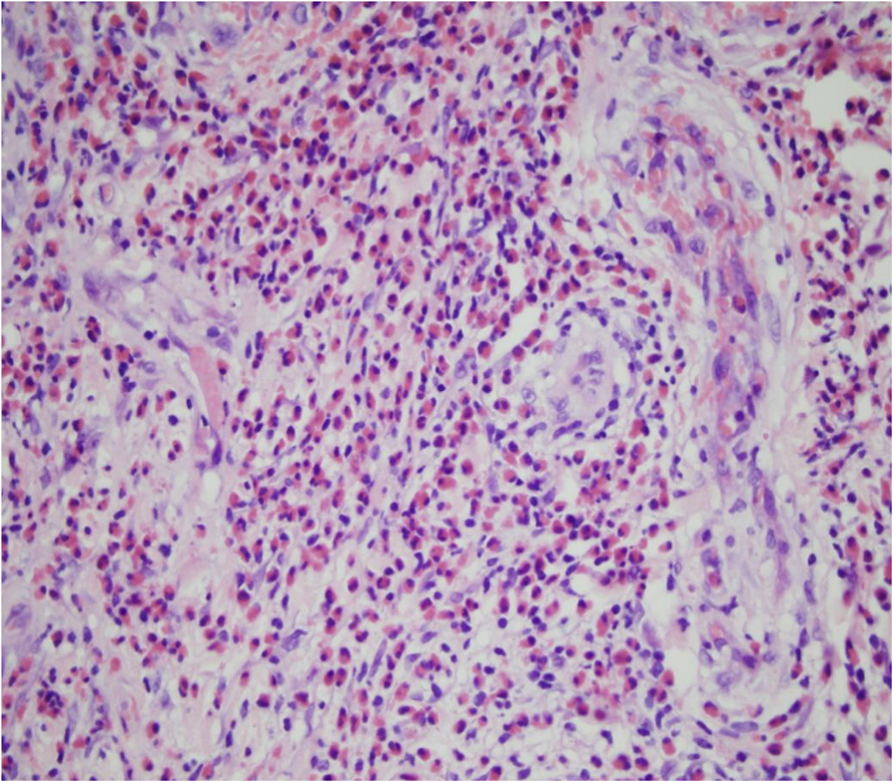
Fibrinoid degeneration and the infiltration consisting of numerous eosinophils and a few neutrophils perivascular and into vessel walls (H&E × 400).

Additional laboratory analysis revealed: white blood cell count 11.8×10^9^/l with 28.7% eosinophils (3.4 × 10^9^/l; normal 0.05-0.5 × 10^9^/l); elevated erythrocyte sedimentation rate (32 mm/h; normal 0–15 mm/h); elevated C-reactive protein level (14.5 mg/1; normal 0–8.2 mg/1); negative antistreptolysin O titer and rheumatoid factor; elevated serum immunoglobulin E level (658.3 IU/ml; normal 0–100 IU/ml); normal serum immunoglobulins G, M, and A levels; normal liver enzyme level; abnormal alkaline phosphatase (131 U/l; normal 26–117 U/l); negative anti-neutrophil cytoplasmic antibodies(ANCA; MPO-ANCA 9 Ru/ml; normal 0–20 Ru/ml. PR3-ANCA 7 Ru/ml; normal 0–20 Ru/ml.); negative antinuclear; negative HIV and syphilis antibodies; normal hepatitis A, B, and C serology; hemolytic complement results consistent with inflammation (C3 1260 mg/1; normal 900–1800 mg/1, C4 430 mg/1; normal 100–400 mg/1); normal urine analysis; and stool examinations negative for parasites and ova. A skin biopsy was performed from a purpuric angioedema lesion on the right limb, providing a specimen of about 1.0 cm × 0.5 cm × 0.7 cm. It revealed a normal epidermis and an infiltration consisting of numerous eosinophils and a few neutrophils perivascular, into vessel walls, in the upper and deep dermis, and in the subcutaneous tissue. Thickening of the vessel walls, numerous extravascular erythrocytes, fibrin thrombi in the lumens, and fibrinoid degeneration (Figures 2, 3, 4, 5) were noted. The diagnosis of RCNEV was made.

The patient was initially treated with 1 mg/kg prednisone daily, compound glycyrrhizin 150 mg daily. Mupirocin ointment was applied to the necrotic lesions. Immediate improvements in clinical manifestations and inflammation were noted. After 2 days, intensive pruritus and angioedema decreased rapidly. After 7 days, pruritus and angioedema had disappeared, and laboratory examinations showed that white blood cell and eosinophil cell count had normalized. One month later, the necrotizing lesions healed, with some remaining as superficial scars. The dose of prednisone was then slowly decreased. All hematological and biochemical examinations were normal and no new lesions were noticed after 4 months of follow-up. As any attempts to discontinue prednisone always result in recurrence [1], the patient presently continues to take prednisone at a dose of 10 mg daily.

## Discussion

Chen et al. [2] reported one male and two female patients diagnosed with RCNEV. The male patient, a 17-year-old in 1973, noted purpuric lesions on his feet and buttocks, which later involved his entire body. One of the two females was a 56-year-old woman in 1989, and presented with pruritic purpuric lesions of 6-month duration. Another woman, at the age of 18 years in 1967, presented with gingivitis, pruritic, erythematous, and purpuric papules, and angioedema of the hands. Skin biopsy specimens showed a common feature: necrotizing vasculitis with marked perivascular eosinophil infiltration. In 2000, Launay et al. [3] reported one patient who was an 81-year-old woman in 1996 who presented with intensively itchy, infiltrating, necrotic purpuric lesions on the lower limbs for 2 weeks. Skin biopsy was performed on a necrotizing lesion which showed necrotizing vasculitis in the deep dermis with striking infiltration by eosinophils. In 2007, Tanglertsampan et al. [1] reported a 53-year-old white male diagnosed with RCNEV. He was treated with indomethacin, with a favorable response.

The pathogenesis of RCNEV is not fully understood, but cytotoxic eosinophil granule proteins such as major basic proteins are found to deposit in the areas of blood vessels, suggesting that eosinophils mediate vascular damage in this disease process [3,4]. Eosinophils release IL-5, C4, and platelet-activating factor, which may lead to increased vascular permeability and induce cutaneous lesions such as purpuric papules and angioedema [5,6]. Although he had more than five years history of psoriasis, no evidence confirmed a correlation between psoriasis and RCNEV.

A review of the literature has demonstrated that peripheral blood eosinophilia is related to many dermatoses such as hypereosinophilic syndrome(HES), wells syndrome,or eosinophilic cellulitis, Churg-Strauss syndrome (CSS) and Eosinophilic fasciitis (EF) [7-13]. Clinico-pathological features of these diseases are listed in Table 1.

**Table 1:** Clinicopathological features of HES, Wells syndrome, CSS and EF HES

	**HES**	**Wells syndrome**	**CSS**	**EF**
**Sex ratio (M:F)**	**9.1**	**1:1**	**1:1**	**2:1**
Age	HES can happen at any age, although it is more common in adults	Wells syndrome usually affects adults	CSS can present at any age, with the mean age of onset being 40 years	EF usually affects adults between 20 and 60 years
Clinical features	Skin rashes such as urticaria or angioedema	Urticaria, cellulitis, annular plaques vesiculo-bullous lesions and edema	Hypereosinophilia, asthma, pulmonary infiltrates, and clinical evidence of vasculitis	Similar to scleroderma or systemic sclerosis
Histopathological features	Eosinophilic infiltration with few lymphocytes, perivascular infiltration in dermis region, but not true necrotizing vasculitis	Eosinophilic infiltrates and flame figures in the absence of vasculitis. perivascular eosinophilic infiltration in the dermis, but not true necrotizing vasculitis	Peripheral blood eosinophils increase significantly, and neutrophil-rich leukocytoclastic vasculitis and granulomatous	Numerous inflammatory infiltrations of lymphocytes and eosinophils within the fascia
Treatment	Prednisone, hydroxyurea, chlorambucil and vincristine.	Corticosteroids, calcineurin inhibitors, griseofulvin, H1 antihistamines, cyclosporine, dapsone	Prednisolone, azathioprine and cyclophosphamide	Prednisone
Clinical outcome	More than 80% of HES patients survive five years or more	It tends to resolve in weeks or months, usually without scarring. It occasionally recurs. In these recurrent cases, it can take years to ultimately resolve	The mean five years mortality rate is 28%	The prognosis is usually good in the case of an early treatment if there is no visceral involvement

In our case, not only an exclusive clinical histopathological feature of fibrinoid degeneration of small vessel walls, but also necrotizing vasculitis of dermal small vessels with prominent infiltration of eosinophils strongly supported the diagnosis of RCNEV.

With regard to the treatment of RCNEV, systemic corticosteroids are most commonly selected because of their effectiveness. Immediate improvements in clinical manifestations and inflammation were noted in our patient following the administration of prednisone. However, some measures should be taken to avoid its side effects if it is used persistently. Compound glycyrrhizin can reduce the activity of T-lymphocyte subset and play an anti-inflammatory role [14]. Mupirocin ointment was used to prevent or control local infection of necrotic lesions. This therapeutic regimen proved to be suitable in our patient, and we consequently obtained a good clinical outcome.

## Conclusion

RCNEV is rare and has clinical and histopathological features, which are different to other conditions of hypereosinophilia and eosinophilic vasculitis. Systemic corticosteroid is very effective in the treatment of RCNEV.

## Consent

Written informed consent was obtained from the patient for publication of this Case Report and any all accompanying images. A copy of the written consent is available for review by the Editor-in-Chief of this journal.

## Abbreviations

RCNEV: Recurrent cutaneous necrotizing eosinophilic vasculitis; ANCA: Anti-neutrophil cytoplasmic antibodies; MPO: Myeloperoxidase; PR3: Proteinase 3; HES: Hypereosinophilic syndrome; CSS: Churg-Strauss syndrome; EF: Eosinophilic fasciitis.

## Competing interest

The authors declare that they have no competing interests.

## Authors’ contributions

WFL designed the study, performed the histological evaluation, wrote the paper; FRZ and XML participated histological diagnosis; WC was involved in literature search and preparing the material. HYS and YXC participated in providing the clinical information of this case. All authors read and approved the final manuscript.
